# Supplementary material for: Robust immune response to COVID-19 vaccination in the island population of Greenland
Source: Commun Med (Lond). 2024 Sep 6;4:173. doi: 10.1038/s43856-024-00602-y (PMC11379896; doi:10.1038/s43856-024-00602-y)
Supplement: Supplementary file 2 — Supplementary Information [file 43856_2024_602_MOESM2_ESM.pdf]

Supplementary information

Supplementary Note 1. Participant enrollment.

Potential participants were contacted by three different approaches. First, a sorted list from the Greenlandic Electronic Medical Patient Registry, COSMIC, containing data on vaccination status and contact information that was provided by the Health Authorities. Possible participants were then contacted by telephone and offered participation. Second, participants were recruited by personal approach at various locations such as shopping malls, workplaces, COVID vaccination facilities, or the University of Greenland. Third, social media platforms, local and national newspapers, radio, and television were used to advertise the study and to recruit volunteers who met the inclusion criteria. There were no exclusion criteria.

Supplementary Fig. 1. *Top:* Illustration of the COVID-19 pandemic course in Greenland, highlighting the specific time points of sample collection and vaccine dose distribution. *Bottom:* Blood samples were collected at Time 1, approximately 2 months (median 9 weeks, IQR 8-13 weeks) after the second vaccine dose, and at Time 2, approximately 11 months (median 45 weeks, IQR 44-48 weeks) after the second vaccine dose. Saliva samples and blood samples for measurement of T-cell response were only collected at Time 2. Dotted lines indicate the Interquartile Range (IQR, Q1-Q3) and median for the interval between blood sampling and the second vaccine dose.

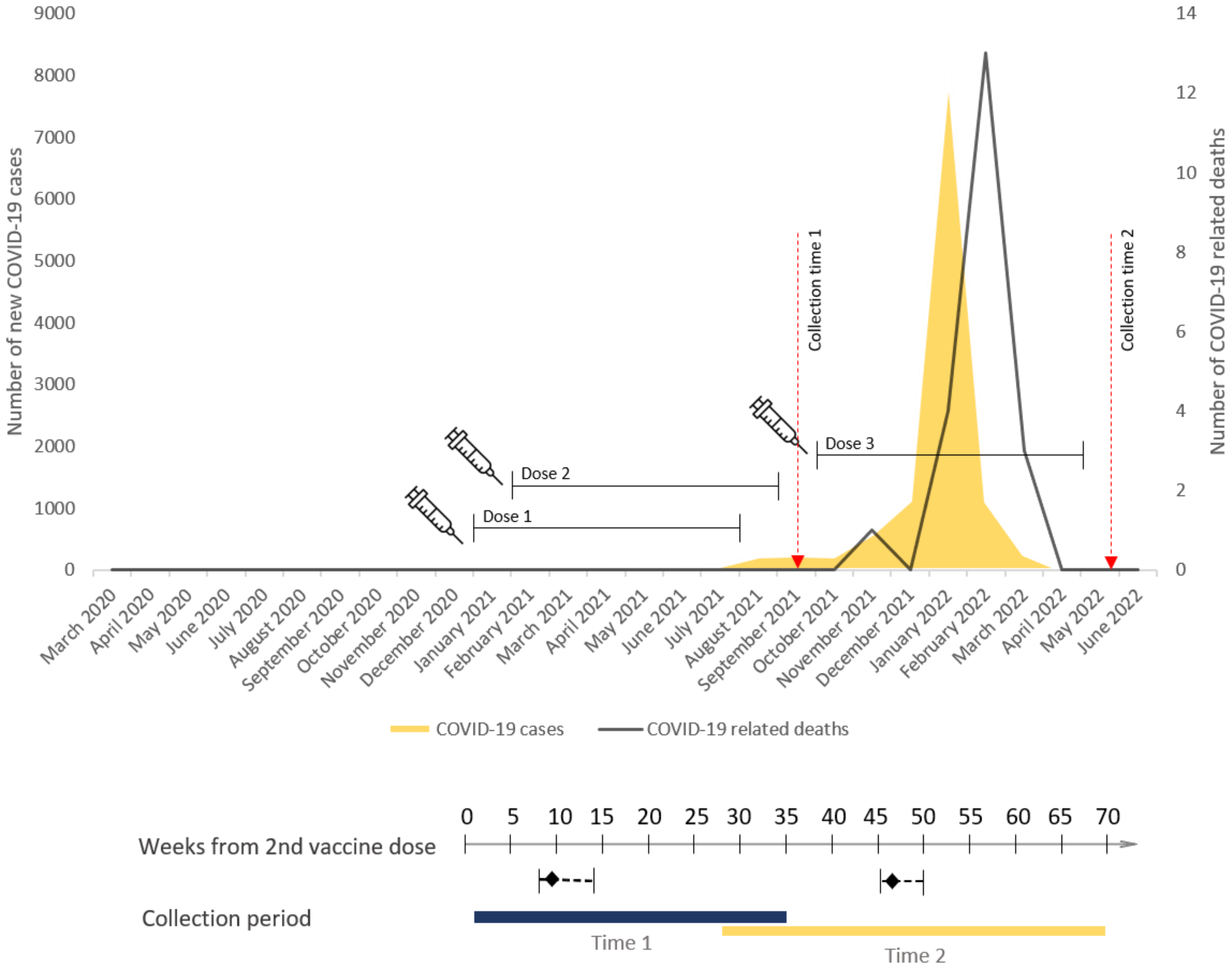

Figure reproduced after Noahsen, P. *et al.* The COVID-19 pandemic in Greenland, epidemic features and impact of early strict measures, March 2020 to June 2022. *Euro Surveill.* **28**, (2023).

Data source: WHO COVID-19 Dashboard (Greenland: WHO Coronavirus Disease (COVID-19) Dashboard)

Supplementary Table 1. Summary statistics of total Ig anti-spike glycoprotein antibody levels in plasma (measured in kilobinding antibody units per litre, kBAU/L) across different explanatory variables. The table includes medians and interquartile ranges (IQR, Q1-Q3).

**Variables**		**Median (IQR)**		
	**N**	**Time 1 (2 months after second vaccine dose)**	**N**	**Time 2 (11 months after second vaccine dose)**
Age				
**20-29 years**	62	4293 (157, 16073)	*34*	16523 (2777, 25000)
**30-39 years**	106	4212 (32, 25000)	*77*	15838 (2926, 25000)
**40-49 years**	57	3667 (179, 25000)	*51*	18779 (1714, 25000)
**50-59 years**	131	2493 (92, 25000)	*99*	17712 (135, 25000)
**60 years or above**	71	1689 (32, 25000)	*59*	11492 (152, 25000)
Charlson Comorbidity Score Index				
**0**	*353*	3282 (32, 25000)	*266*	16252 (135, 25000)
**1**	*59*	3022 (32, 25000)	*45*	14385 (204, 25000)
**2 or more**	*18*	1511 (127, 5929)	*12*	9943 (2180, 25000)
Gender				
**Female**	*274*	3307 (32, 25000)	*207*	16203 (135, 25000)
**Male**	*156*	3052 (32, 25000)	*116*	15412 (152, 25000)
Ethnicity				
**Inuit**	*292*	3212 (32, 25000)	*218*	16069 (135, 25000)
**Mixed**	*120*	3035 (32, 25000)	*91*	15533 (204, 25000)
**Non-Inuit**	*18*	3192 (593, 7627)	*14*	16912 (152, 25000)
Vaccine type				
**mRNA-1273 (Moderna)**	*415*	3267 (32, 25000)	*13*	15942 (135, 25000)
**BNT162b2 (Pfizer/BioNTech)**	*15*	320 (32, 25000)	*10*	14675 (221, 25000)

Supplementary Fig. 2. Comparison of the total plasma immunoglobulin (Ig) specific to the anti-spike glycoprotein (measured in kilobinding antibody units per litre, kBAU/L) levels between individuals who received the mRNA1273 (Moderna) and BNT162b2 (Pfizer/BioNTech) vaccines, grouped by sample collection time. Time 1 (2 months after second vaccine dose) and Time 2 (11 months after second vaccine dose). The boxplots present the lower quartile, median, and upper quartile, and the error bars indicate 95% CI. Wilcoxon-Mann-Whitney test was performed to compare vaccines. P-values < 0.05 were considered significant.

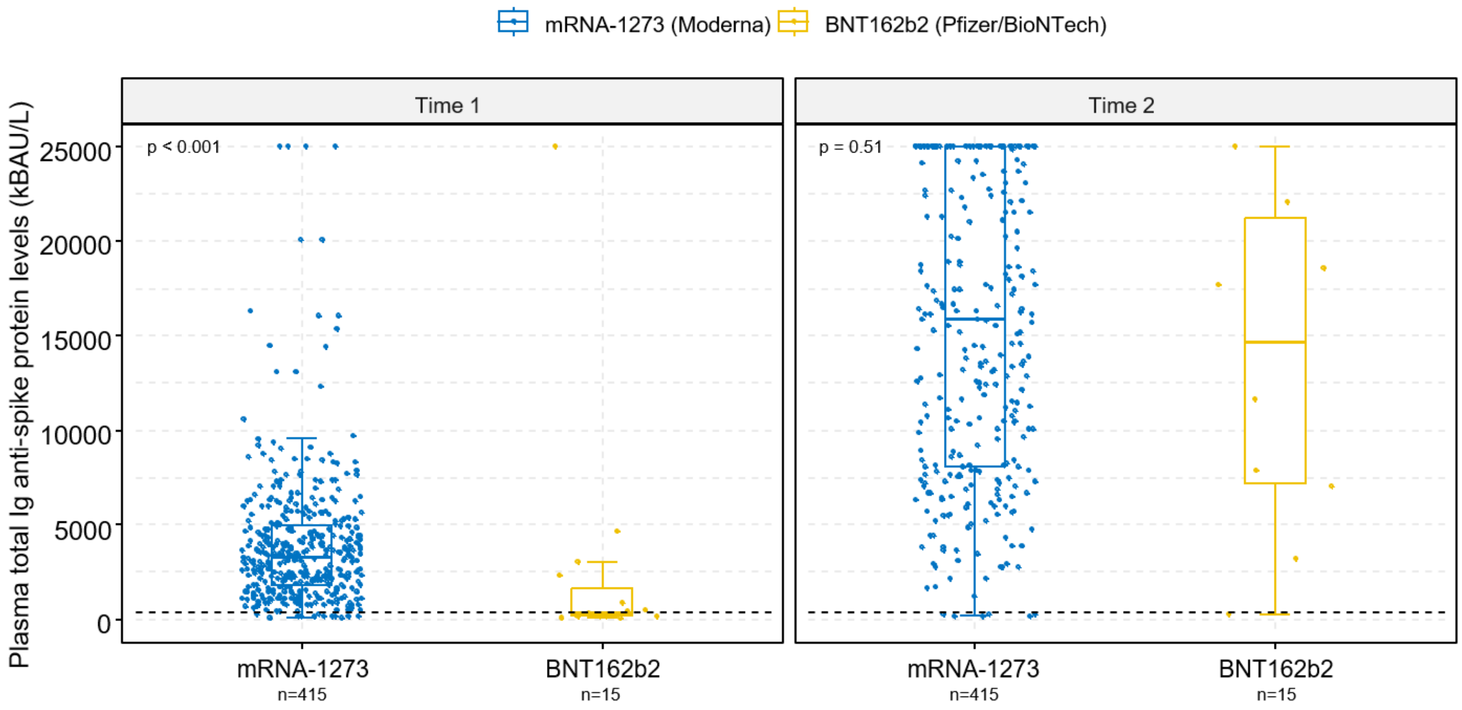

Supplementary Table 2. Unadjusted and age-adjusted estimates from linear regression models with 95% confidence interval (CI) for the logarithm (log10) of the antibody response in saliva (total Ig anti-spike glycoprotein measured in kilobinding antibody units per litre, kBAU/L) at Time 2 (11 months post-primary vaccination) by explanatory variables. The outcome is defined as the relative change in antibody level compared with the reference group, presented as exponentiated coefficients with corresponding 95% CI. P-values < 0.05 were considered significant.

		**Model 1[a]**		**Model 2[b]**	
**Variables**	**N**	**Estimate (95% CI)**	**P-value**	**Estimate (95% CI)**	**P-value**
**Gender**					
Female	69	Reference	Reference	Reference	Reference
Male	36	1.16 (0.97, 1.39)	0.096	1.17 (0.97, 1.41)	0.097
**Age**					
20-29 years	10	Reference	Reference	-	-
30-39 years	26	0.90 (0.65, 1.26)	0.535	-	-
40-49 years	20	0.91 (0.64, 1.28)	0.578	-	-
50-59 years	27	0.85 (0.61, 1.19)	0.342	-	-
60+ years	21	0.88 (0.63, 1.24)	0.470	-	-
**Ethnicity**					
Inuit	73	Reference	Reference	Reference	Reference
Mixed	28	1.06 (0.87, 1.29)	0.540	1.08 (0.88, 1.34)	0.462
Non-Inuit	4	0.87 (0.55, 1.36)	0.538	0.87 (0.55, 1.40)	0.571
**Previous SARS-CoV-2 infection**					
No	29	Reference	Reference	Reference	Reference
Yes	76	1.74 (1.49, 2.04)	< 0.001	1.76 (1.50, 2.08)	< 0.001

[a]unadjusted
[b]adjusted for age

Supplementary Table 3. Unadjusted and age-adjusted estimates from linear regression models with 95% confidence interval (CI) for the logarithm (log10) of the $CD4^{+}$ and $CD4^{+}$+$CD8^{+}$ T-cells IFN-γ release levels (measured in International Unit per millilitre, IU/mL) at Time 2 (11 months post-primary vaccination) by explanatory variables. The outcome is defined as the relative change in IFN-γ release levels compared to the reference group, presented as exponentiated coefficients with corresponding 95% CI. P-values < 0.05 were considered significant.

		**$CD4^{+}$ T-cells**				**$CD4^{+}$+$CD8^{+}$ T-cells**			
		**Model 1[a]**		**Model 2[b]**		**Model 1[a]**		**Model 2[b]**	
**Variables**	**N**	**Estimate (95% CI)**	**P-value**	**Estimate (95% CI)**	**P-value**	**Estimate (95% CI)**	**P-value**	**Estimate (95% CI)**	**P-value**
**Gender**									
Female	51	Reference	Reference	Reference	Reference	Reference	Reference	Reference	Reference
Male	27	1.05 (0.80, 1.38)	0.732	1.06 (0.79, 1.42)	0.680	0.94 (0.73, 1.21)	0.613	0.96 (0.74, 1.24)	0.747
**Age**									
20-29 years	9	Reference	Reference	-	-	Reference	Reference	-	-
30-39 years	17	0.90 (0.58, 1.41)	0.639	-	-	0.75 (0.51, 1.10)	0.131	-	-
40-49 years	12	0.78 (0.50, 1.22)	0.261	-	-	0.67 (0.46, 0.97)	0.036	-	-
50-59 years	20	0.97 (0.63, 1.48)	0.875	-	-	0.83 (0.57, 1.21)	0.334	-	-
60+ years	19	0.89 (0.59, 1.35)	0.576	-	-	0.64 (0.45, 0.91)	0.015	-	-
**Ethnicity**									
Inuit	54	Reference	Reference	Reference	Reference	Reference	Reference	Reference	Reference
Mixed	20	1.14 (0.82, 1.58)	0.421	1.20 (0.86, 1.69)	0.282	1.05 (0.78, 1.41)	0.751	1.18 (0.88, 1.59)	0.267
Non-Inuit	4	1.20 (0.60, 2.40)	0.592	1.36 (0.66, 2.81)	0.395	1.19 (0.63, 2.25)	0.583	1.46 (0.78, 2.72)	0.227
**Previous SARS-CoV-2 infection**									
No	19	Reference	Reference	Reference	Reference	Reference	Reference	Reference	Reference
Yes	59	1.25 (0.91, 1.72)	0.159	1.29 (0.93, 1.81)	0.127	1.14 (0.85, 1.53)	0.358	1.19 (0.89, 1.60)	0.228

[a]unadjusted
[b]adjusted for age
