# Supplementary material for: Robust immune response to COVID-19 vaccination in the island population of Greenland
Source: Commun Med (Lond). 2024 Sep 6;4:173. doi: 10.1038/s43856-024-00602-y (PMC11379896; doi:10.1038/s43856-024-00602-y)
Supplement: Supplementary file 3 — Description of Additional Supplementary Files [file 43856_2024_602_MOESM3_ESM.pdf]

Description of Additional Supplementary Files

File name: Supplementary Data 1

File description: primary vaccination, blood sampling and age with effect on the total S-Ab response at Time

File name: Supplementary Data 2-7

File description: Unidentifiable source data
