# Supplementary material for: Robust immune response to COVID-19 vaccination in the island population of Greenland
Source: Commun Med (Lond). 2024 Sep 6;4:173. doi: 10.1038/s43856-024-00602-y (PMC11379896; doi:10.1038/s43856-024-00602-y)
Supplement: Supplementary file 11 — Reporting Summary [file 43856_2024_602_MOESM11_ESM.pdf]

nature portfolio

Corresponding author(s): Mie Møller

Last updated by author(s): Aug 27, 2024

Reporting Summary

Nature Portfolio wishes to improve the reproducibility of the work that we publish. This form provides structure for consistency and transparency in reporting. For further information on Nature Portfolio policies, see our Editorial Policies and the Editorial Policy Checklist.

Statistics

For all statistical analyses, confirm that the following items are present in the figure legend, table legend, main text, or Methods section.

n/a	Confirmed	
☐	☒	The exact sample size (*n*) for each experimental group/condition, given as a discrete number and unit of measurement
☐	☒	A statement on whether measurements were taken from distinct samples or whether the same sample was measured repeatedly
☐	☒	The statistical test(s) used AND whether they are one- or two-sided<br>*Only common tests should be described solely by name; describe more complex techniques in the Methods section.*
☐	☒	A description of all covariates tested
☒	☐	A description of any assumptions or corrections, such as tests of normality and adjustment for multiple comparisons
☐	☒	A full description of the statistical parameters including central tendency (e.g. means) or other basic estimates (e.g. regression coefficient) AND variation (e.g. standard deviation) or associated estimates of uncertainty (e.g. confidence intervals)
☐	☒	For null hypothesis testing, the test statistic (e.g. F, t, r) with confidence intervals, effect sizes, degrees of freedom and P value noted<br>*Give P values as exact values whenever suitable.*
☒	☐	For Bayesian analysis, information on the choice of priors and Markov chain Monte Carlo settings
☒	☐	For hierarchical and complex designs, identification of the appropriate level for tests and full reporting of outcomes
☐	☒	Estimates of effect sizes (e.g. Cohen's d, Pearson's r), indicating how they were calculated

Our web collection on statistics for biologists contains articles on many of the points above.

Software and code

Policy information about availability of computer code

Data collection	No software was used.
Data analysis	All analyses were conducted using R, version 4.2.3.

For manuscripts utilizing custom algorithms or software that are central to the research but not yet described in published literature, software must be made available to editors and reviewers. We strongly encourage code deposition in a community repository (e.g. GitHub). See the Nature Portfolio guidelines for submitting code & software for further information.

Data

Policy information about availability of data

All manuscripts must include a data availability statement. This statement should provide the following information, where applicable:

- Accession codes, unique identifiers, or web links for publicly available datasets
- A description of any restrictions on data availability
- For clinical datasets or third party data, please ensure that the statement adheres to our policy

Limitations apply to data accessibility. Due to Greenlandic law and ethical considerations, the full dataset is not publicly available because it contains highly detailed and individually linked data. Unidentifiable source data is available in Supplementary Data files 2-7. To access raw data, researchers are required to obtain ethics approval from the Greenland Research Ethics Committee and consent from study participants. Contact the corresponding author for further details on these limitations and the specific conditions for accessing raw data.

Human research participants

Policy information about studies involving human research participants and Sex and Gender in Research.

Reporting on sex and gender	We used the term 'gender' in our study, determined through self-reporting via questionnaires. All participants provided informed consent (both written and oral) at enrollment, during which they completed the questionnaire. Gender-based analyses were conducted using regression models to assess associations between gender and the vaccine-induced humoral and/or cellular antibody response.
Population characteristics	Of the entire cohort; 63.7% were females, 68.0% of Inuit descent, with a median age of 46.5 (IQR 33–57) years, 82% with a Charlson Comorbidity Score Index of 0, and a BMI median of 27.7 (IQR 25.1-31.5).<br>95.1% were vaccinated with the mRNA-1273 vaccine (Moderna), while 3.5% received the BNT162b2 vaccine (Pfizer–BioNTech).
Recruitment	We used a mixed recruitment approach involving random selection and voluntary participation. The study participants were recruited using different approaches including telephone contact from a list of vaccinated individuals living in Nuuk/Illulissat, personal approaches in public spaces and workplaces, vaccination centers, and the utilization of social media, local radio, and newspapers to advertise the study and encourage enrollment.<br>Only adults (>18 years), living in Nuuk or Ilulissat, Greenland were included. All participants had recieved two doses of a SARS-CoV-2 mRNA vaccine at the time of enrollment.<br>Selection bias may be present as we only recruited individuals living in the two largest cities of Greenland, where the population can differ significantly from those in smaller villages. Although we are unsure if this bias influenced our results, the vaccine-induced antibody response is likely not influenced by culture and environment, minimizing its potential effect.<br>Information bias is also possible since some data relies on self-reporting, such as previous SARS-CoV-2 infection and/or booster vaccination. Consequently, there is some uncertainty regarding the impact of these parameters on our results.
Ethics oversight	Greenland Science Ethics Committe

Note that full information on the approval of the study protocol must also be provided in the manuscript.

Field-specific reporting

Please select the one below that is the best fit for your research. If you are not sure, read the appropriate sections before making your selection.

☒ Life sciences ☐ Behavioural & social sciences ☐ Ecological, evolutionary & environmental sciences

For a reference copy of the document with all sections, see nature.com/documents/nr-reporting-summary-flat.pdf

Life sciences study design

All studies must disclose on these points even when the disclosure is negative.

Sample size	Sample size was not predicted in advance. However, during the actual implementation of the study, we enrolled 430 (~1 % of the Greenlandic adult population) participants which we believed was sufficient to demonstrate a general and representative humoral and cellular antibody response in Greenlanders.
Data exclusions	No data was excluded.
Replication	When measuring the vaccine-induced antibody response, we utilized two different assays: one measuring the total immunoglobulin response and another measuring the IgG-specific response. This approach served to showcase the quality of the two assays and ensure the reproducibility of our results. The measurements from the two assays correlated positively; consequently, we opted for the simpler and more cost-effective assay to present our results.
Randomization	There was no randomization in this study, given that no experimental work was performed. However, the study participants were randomly recruited using various approaches (see the above). The criterion of enrollment in the study was adults, born and living in Nuuk or Ilulissat, who had been vaccinated (2 doses) with an mRNA-vaccine and volunteered to participate.
Blinding	Blinding was not applicable to the study as it was not an intervention or experimental study.

Reporting for specific materials, systems and methods

We require information from authors about some types of materials, experimental systems and methods used in many studies. Here, indicate whether each material, system or method listed is relevant to your study. If you are not sure if a list item applies to your research, read the appropriate section before selecting a response.

Materials & experimental systems

n/a	Involved in the study
☐	☒ Antibodies
☒	☐ Eukaryotic cell lines
☒	☐ Palaeontology and archaeology
☒	☐ Animals and other organisms
☒	☐ Clinical data
☒	☐ Dual use research of concern

Methods

n/a	Involved in the study
☒	☐ ChIP-seq
☒	☐ Flow cytometry
☒	☐ MRI-based neuroimaging

Antibodies

Antibodies used

Total (IgG+A+M) SARS-CoV-2 spike glycoprotein antibody (S-Ab) and nucleoprotein antibody (N-Ab) levels were measured using ECLIA Assays (Roche Diagnostics, Mannheim, Germany).

Validation

The SARS-CoV-2 nucleoprotein antibody have for instance been validated by
Harritshøj, L. H. et al. Comparison of 16 serological SARS-CoV-2 immunoassays in 16 clinical laboratories. J. Clin. Microbiol. 59, (2021).

The SARS-CoV-2 spike glycoprotein immunoassays have among others been validated by
Whitaker, H. J. et al. Nucleocapsid antibody positivity as a marker of past SARS-CoV-2 infection in population serosurveillance studies: impact of variant, vaccination, and choice of assay cut-off. medRxiv 2021.10.25.21264964 (2021) doi:10.1101/2021.10.25.21264964.
Infantino M, Pieri M, Nuccetelli M, et al. The WHO International Standard for COVID-19 serological tests: towards harmonization of anti-spike assays. Int Immunopharmacol 2021; 100: 108095.
Ferrari D, Clementi N, Spano SM, et al. Harmonization of six quantitative SARS-CoV-2 serological assays using sera of vaccinated subjects. Clin Chim Acta 2021; 522: 144-51.
